# Toxic effects of fenitrothion on freshwater microcosms in Bangladesh

**DOI:** 10.1016/j.toxrep.2020.12.002

**Published:** 2020-12-04

**Authors:** Mohammad Shadiqur Rahman, Kizar Ahmed Sumon, Md Jasim Uddin, Md Shahjahan

**Affiliations:** Laboratory of Fish Ecophysiology, Dept. of Fisheries Management, Bangladesh Agricultural University, Mymensingh, 2202, Bangladesh

**Keywords:** Organophosphate insecticide, Macro-invertebrates, Phytoplankton and zooplankton

## Abstract

The organophosphate pesticide fenitrothion is widely used as an agricultural pesticide to control tiger bug in larval rearing for aquaculture. The objectives of the present study were to assess the effects of fenitrothion on certain structural (phytoplankton, zooplankton, macro-invertebrates and periphyton) and functional (organic matter decomposition) endpoints of freshwater microcosms. Fenitrothion 50 EC was applied in 12 microcosms (PVC tanks having 400 L of dechlorinated tap water) providing concentrations of 0, 25, 50 and 100 μg/L at a 4-day interval over a period of 4 weeks. Each of the experimental treatment was executed in three replicates. The results indicated the consistent significant effects for most of the species composition of zooplankton and macro-invertebrates. Univariate analysis showed a significant decrease in abundance (p < 0.05) of all identified insects (i.e. *Notonecta* sp., *Gerris* sp., *Ranatra linearis* and Chironomid larvae), when compared to control in all sampling days throughout the treatment period (no observed effect concentration; NOEC = < 25 μg/L). No consistent significant effects were observed for most of the phytoplankton taxa and organic matter decomposition and water quality variables (dissolved oxygen, free CO_2_, pH, nitrate etc.). However, several taxa of different endpoints were found sensitive to even the lowest concentration of fenitrothion (25 μg/L). Further studies with acute and chronic conditions are recommended involving more local species exposed to < 25 μg/L of fenitrothion.

## Highlights

• Effects of fenitrothion on community level and functional endpoints of freshwater ecosystems in microcosms were studied.
• No consistent significant effects were observed for most of the phytoplankton taxa.
• There was a significant decrease for most of the species composition of zooplankton and macro-invertebrates.
• Several taxa were sensitive to even the lowest concentration of fenitrothion.

## Graphical abstract

**Figure:**
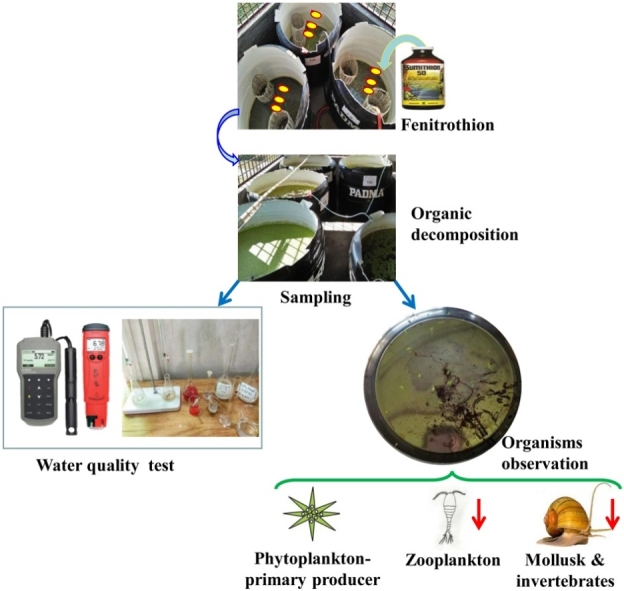

## 1 Introduction

Agricultural intensifications are inevitable to fulfill the food and nutritional demand of burgeoning population coping with ever reducing available arable land in the world [1]. Further, natural calamities including sudden flood, seasonal water scarcity, salinity intrusion into coastal land, and cyclones and storms are constant threats for agricultural crop production which limit may the agricultural intensification. While dealing with these adversities and for progressing the agricultural production, farmers cultivate various high-yielding cultivars of different crops. But these high-yielding cultivars are most vulnerable to diseases and pests [2]. Therefore, the uses of various pesticides in agricultural land to control pests as well as to increase crops output per acre of land is a common practice in Bangladesh. Furthermore, the agricultural administration also inspires the farmers to use pesticides to increase the output per acre of land through providing subsidy [3].

In Bangladesh, nearly 84 pesticides are registered with 242 trade names under various chemical groups, such as organophosphate, organochlorine, carbamates and pyrethroids to protect various crops [4]. Among them, the use of organophosphate group has become increasingly popular in most of the developing countries like Bangladesh [[5], [6], [7]]. Fenitrothion, a type II organophosphate synthetic pesticide, is extensively used in agriculture to protect various crops including cereals, cotton, rice, vegetables and top fruits [8]. This pesticide was first introduced in 1959 by Sumitomo Chemical Company and Bayer Leverkusen and later by American Cyanamid Company [9].

The primary purpose of using fenitrothion is to remove tiger bug prior to stocking fish larvae in fish ponds. Moreover, the use of fenitrothion in agriculture may reach to the aquatic environments through direct spray, runoff, leaching, and disposal and washing of containers and equipment in water [6]. After reaching into the waterbodies, fenitrothion may affect the non-target aquatic organisms belonging to different trophic levels, when exceeding the threshold level [6]. Due to its’ potential toxic effects, it is recommended that formulations containing fenitrothion as an active ingredient must have the signal word “caution” on their label [10].

In the past, a number of studies have been conducted in assessing the effects of fenitrothion on non-target aquatic organisms. Most of the studies have focused on the single species laboratory tests on phytoplankton [11,12] zooplankton [[13], [14], [15], [16]], macro-invertebrates [9,[17], [18], [19]] and fish [10,[20], [21], [22], [23], [24], [25], [26], [27], [28]] using sumithion as a test compound. Sabater and Carrasco [11] estimated the 96-h no observed effect concentration (NOEC) values of fenitrothion for *Chlorella* v*ulgaris* and *Chlorella saccharophila* to be 4600 and 1700 μg/L, respectively. Leboulanger et al. [29] estimated the 48-h EC_50_ (1840 μg/L) of fenitrothion for one of the copepods *Mesocyclops* spp. In fish, fenitrothion altered blood parameters and histopathology of different organs [22,[21], [22], [23], [24], [25], [26], [27], [28]].

Over the past decades, model ecosystem studies, such as microcosms and mesocosms are using as important techniques to assess the risk of pesticides [[30], [31], [32], [33]] and veterinary medicines [34]. There are a number of advantages of using microcosm for toxicity studies, i.e. microcosms allow replications, experimental set-up and ecological realism in a controlled environment [35].

To date, two microcosm studies have been performed to elucidate the toxic effects of fenitrothion on the plankton [15] and soil microorganisms [36]. However, there is paucity of information in different parts of the world focusing on the effects of fenitrothion on both structural (phytoplankton, zooplankton, macro-invertebrates and periphyton) and functional (organic matter decomposition) endpoints in aquatic ecosystems. Hence, the present study aimed at assessing the effects of fenitrothion on certain structural and functional endpoints of freshwater ecosystems in Bangladesh.

## 2 Materials and methods

### 2.1 Experimental design

The materials and methods of this study were followed a study described by Sumon et al. [33]. This study was conducted in twelve freshwater microcosms at the laboratory of fish Ecophysiology, Bangladesh Agricultural University, Bangladesh from July to October 2018 (temperature; 25−30 °C). Twelve polyvinyl chloride (PVC) tanks (diameter: 170 cm; total height: 75 cm) were used as microcosms, having four treatments, each with three replications. Each microcosm was filled with 4.5 cm of sediment and 400 L of tap water. Sediment samples were collected from nearby ponds where agricultural activities have not been practiced for many years. To provide sufficient oxygen, an aeration system was installed in each microcosm. Concentrated plankton (equal volumes) and macro-invertebrates (equal numbers) were collected from the ponds where sediment was collected and stocked in each microcosm. The plankton and macro-invertebrates were allowed to establish themselves over a pre-treatment period of 4 weeks prior to sumithion exposure. About 20 % water of the tanks was exchanged every two weeks among the microcosms to homogenize the structure of communities in the systems. Urea (1.4 mg/L) as source of nitrogen and triple super phosphate (0.18 mg/L) as source of phosphorus was used every two weeks in the microcosms during the whole experimental period according to the recommendations described by Daam and Van den Brink [37].

### 2.2 Application of sumithion

Fenitrothion (formulation: fenitrothion; active ingredient: 50 EC; manufacturer: Sumitomo Chemical Company Limited, Japan) was purchased from a local pesticide seller. After 4 weeks of pre-treatment period, fenitrothion was poured and mixed thoroughly in each microcosm at concentrations of 0, 25, 50 and 100 μg/L at 4-day interval over a period of 4 weeks. The stock solution of 1 L was prepared by dissolving the weighed amount in distilled water to get the desired concentration (500 g/L) of fenitrothion.

### 2.3 Plankton sampling

Plankton was sampled on day 1 and day 7 before fenitrothion application, and on days 7, 14, 21 and 28 after start application of fenitrothion. Water samples (5 L) were collected using Perspex tube in a plastic bucket and passed through plankton net with a mesh size of 20 μm for phytoplankton and 55 μm for zooplankton [33]. The 5 L water samples were concentrated to a volume of 100 mL. Subsequently, the concentrated samples were preserved in plastic bottle with 10 % buffered formalin solution and stored at 4 °C until further identification. Three sub-samples (1 mL) of the concentrated phytoplankton and zooplankton samples were analyzed at an inverted microscope (MICROS-MCX100, Austria) with a magnification of 100 × . Phytoplankton and zooplankton were analyzed to the lowest practical level, and the species or genus densities were recalculated as the number of individuals per liter of microcosm water.

### 2.4 Macro-invertebrates sampling

The diversity and abundance of macro-invertebrates were assessed by using two artificial substrates in each microcosm. Bamboo made basket (height- 30 cm and diameter- 20 cm) were used as an artificial substrate. Two baskets were positioned on the sediments in each of the microcosm and they were allowed to have a colonization period of 14 days. Macro-invertebrates were sampled on day 1 and day 7 before application of the fenitrothion and on days 7, 14, 21 and 28 after the first application of fenitrothion. Two baskets were sampled alternately from each of the microcosm. On each sampling date, one of the baskets was lifted from the sediment and directly enclosed by nylon net. The substrate was gently shaken inside the net to collect the invertebrates. Furthermore, the net was passed through the water column next to the tanks’ wall covering approximately one-quarter of the walls’ surface in order to catch swimming macro-invertebrates. The invertebrates (Chironomid larvae and *Tubifex tubifex*) inhabiting into the sediment were collected by a core sediment sampler (inner diameter: around 8 cm) and transferred to a white plastic tray, identified, counted and stocked them into their original microcosms.

### 2.5 Water quality parameters monitoring

Temperature, dissolved oxygen (DO), free CO_2_, pH, total alkalinity, nitrate and phosphate were monitored at 10 a.m. on day 1 and day 7 before the application of fenitrothion, and on days 7, 14, 21 and 28 after the first application of fenitrothion. A digital thermometer, DO meter (Model DO5509, Lutron Taiwan) and a portable pH meter (Model-RI02895, HANNA) were used to measure water temperature, dissolved oxygen and pH, respectively. Free CO_2_ was measured using phenolphthalein indicator and 0.0227 N NaOH titrant, and total alkalinity was measured using methyl orange indicator and 0.02 N H_2_SO_4_ titrant.

### 2.6 Chlorophyll-a

The chlorophyll-a content was measured on day 7, 14, 21 and 28 after first fenitrothion exposure according to the method described by Greenberg et al. [38]. In brief, 50 mL of water sample was filtered in microfilter paper through a vacuum filter. Microfilter paper was cut into small pieces and put into a graduated plastic tube containing 20 mL acetone. The filter paper was mixed well using a tissue homogenizer and wrapped with aluminum foil and kept overnight at 4 °C. On the following day, after thawing at ambient temperature the tube was centrifuged at 3000 rpm for 5 min and the supernatant was taken in the cuvette. Then reading was taken at 664, 647 and 630 mm using UV spectrophotometer and chlorophyll-a content was calculated using the following formula:

Chlorophyll-a (mg/L) = 11.85 (OD 664) – 1.54 (OD 647) – 0.08 (OD 630) [38].

### 2.7 Organic matter decomposition

In the present study, we examined the effects of sumithion on organic matter decomposition (OMD). Four litter bags containing 2 g of banana leaves were placed into each microcosm 1 day before the start of the application of fenitrothion. The banana leaves were dried in oven at 40 °C for 48 h after washed under tap water for 48 h. Then 2 g of dried banana leaves were put in each litter bag and the bags were suspended at 30 cm water depth in the microcosms. After the application of fenitrothion, one litter bag was collected from each microcosm on days 7, 14, 21 and 28. The collected material was weighted after dried in an oven at 40 °C for 48 h. The percentage of OMD was calculated by comparing the final dry weight after the incubation period with the initial dry weight of banana leaves.

### 2.8 Statistical analyses

Statistical analysis of all data was performed by using SPSS software (version 23; SPSS Inc., Chicago, IL, USA). A one-way analysis of variance (one-way ANOVA) and Tukey’s post-hoc test were used to assess the significant differences among the treatments. Differences were considered to be significant if p < 0.05. No observed effect concentrations (NOECs) were calculated as the highest fenitrothion concentration that did not show significant effects as compared to the control [39].

## 3 Results and discussion

### 3.1 Primary producers and chlorophyll-a

**Fig. 1:**
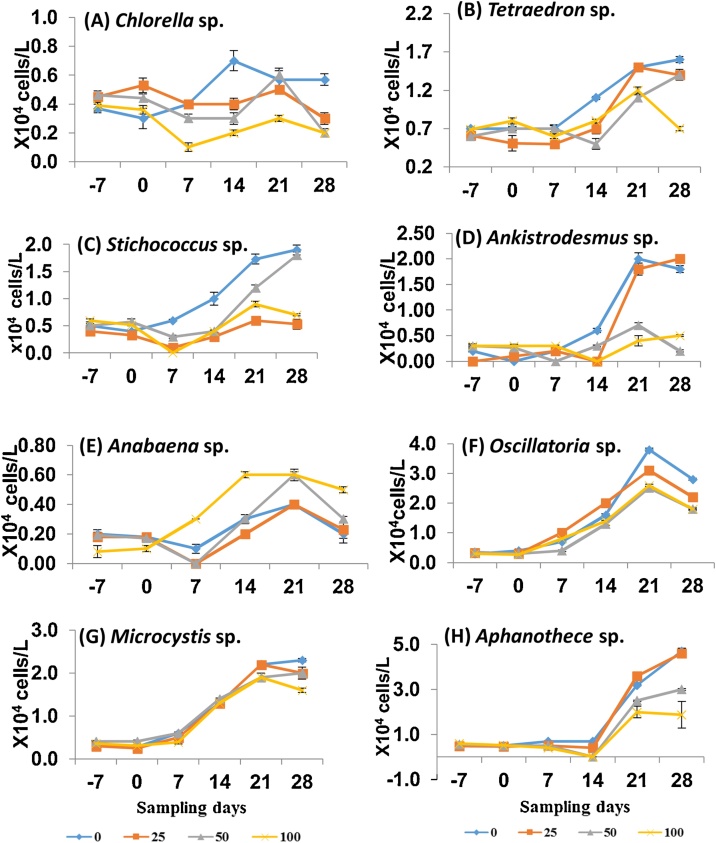
The population dynamics of the phytoplankton taxa; (A) *Chlorella* sp., (B) *Tetraedron* sp., (C) *Stichococcus* sp., (D) *Ankistrodesmus* sp., (E) *Anabaena* sp., (F) *Oscillatoria* sp., (G) *Microcystis* sp. and (H) *Aphanothece* sp. under the four concentrations (0, 25, 50 and 100 μg/L) of fenitrothion. Only the taxa that showed a significant response under treatment period for all sampling days are included.

**Fig. 2:**
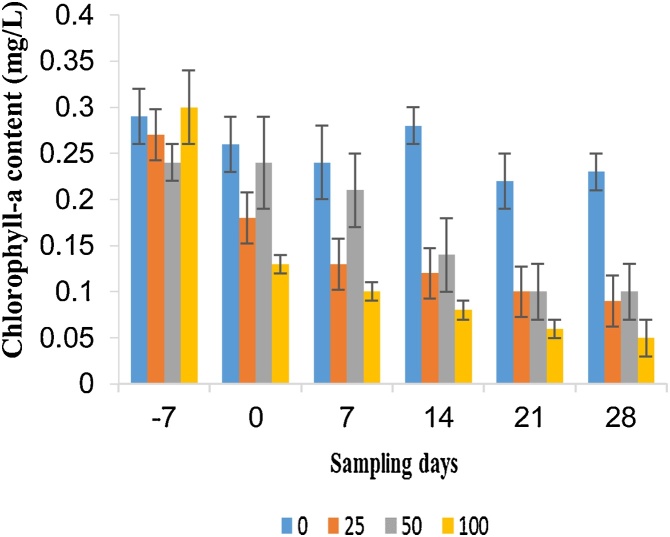
Chlorophyll-a content different sampling days under four concentrations (0, 25, 50 and 100 μg/L) of fenitrothion.

A total of 25 phytoplankton species were identified in the present study. The community of phytoplankton was dominated by Chlorophyceae (13 taxa), followed by Bacillariophyceae (7 taxa), Cyanophyceae (4 taxa) and Euglenophyceae (1 taxon) (Table 1). Among the 13 taxa of Cholorophyceae, only three taxa i.e. *Chlorella* sp., *Tetraedron* sp., and *Stichococcus* sp. were negatively affected from day 7 onwards (Table 1; Fig. 1). There was a significant decrease (p < 0.05) in abundance of *Aphanothece* sp. for all sampling days during the treatment period with a consistent NOEC of ≤ 25 μg/L. In this study, we found the phytoplankton taxa more sensitive to fenitrothion than the previous studies. For instance, Sabater and Carrasco [11] estimated the 96 h NOEC values of fenitrothion for *Chlorella* v*ulgaris* and *Chlorella saccharophila* to be 4600 and 1700 μg/L, respectively; which is much higher than we calculated 7d NOEC (25 μg/L) for *Chlorella* sp. Kent and Currie [40] reported the 96 h EC_50_ value of sumithion for one of Cholorpyceans (*Chlamydomonas segnis*) as 6600 μg/L, which is again two hundred folds higher than our study. On the other hand, photosynthesis is inhibited by more than 75 % by fenitrothion in *Anabaena* and *Aulosira* [41]. Moreover, it has been reported that fenitrothion bio-concentrated (2- to 10-fold) from water to algae [42]. In the present study, results showed consistent significant decrease in chlorophyll-a content from day 14 onwards at all treatments as compared to control (NOEC = <25 μg/L) (Fig. 2).

**Table 1:** No Observed Effect Concentrations (NOECs) for phytoplankton taxa expressed in terms of nominal single-dose of sumithion concentrations (μg/L) measured on each sampling day (One-way ANOVA; p < 0.05).

Endpoints	Sampling days					
	−7	0	7	14	21	28
**Chlorophyceae**						
*Actinestrum* sp.	>	>	25 (+)	50 (-)	>	>
*Chlorella* sp.	>	>	25 (-)	<25 (-)	50 (-)	<25 (-)
*Pediastrum* sp.	>	>	NP	<25 (-)	25 (+)	25 (+)
*Pleurococcus* sp.	>	>	<25 (+)	<25 (-)	<25 (+)	>
*Scenedesmus* sp.	>	>	>	50 (+)	>	<25 (+)
*Spirogyra* sp.	NP	NP	NP	<25 (-)	50 (-)	25 (+)
*Tetraedron* sp.	>	>	50 (-)	<25 (-)	25 (-)	<25 (-)
*Stichococcus* sp.	>	>	<25 (-)	<25 (-)	<25 (-)	50 (-)
*Ulothrix* sp.	NP	25 (+)	NP	<25 (-)	<25 (-)	>
*Cosmerium* sp.	>	>	<25 (-)	50 (-)	50 (-)	>
*Ankistrodesmus* sp.	>	<25 (+)	50 (+)	<25 (-)	25 (-)	<25 (-)
*Volvox* sp.	>	>	>	<25 (-)	<25 (+)	50 (-)
*Closterium* sp.	>	>	>	<25 (+)	>	50 (-)
**Cyanophyceae**						
*Anabaena* sp.	>	>	<25 (-)	50 (+)	25 (+)	50 (+)
*Oscillatoria* sp.	>	>	50 (+)	<25 (-)	<25 (-)	<25 (-)
*Microcystis* sp.	>	>	50 (-)	25 (+)	25 (-)	<25 (-)
*Aphanothece* sp.	>	>	<25 (-)	<25 (-)	<25 (-)	25 (-)
**Bacillariophyceae**						
*Cyclotella* sp.	>	>	>	25 (-)	<25 (-)	<25 (-)
*Fragillaria* sp.	NP	NP	<25 (-)	<25 (-)	>	>
*Navicula* sp.	>	>	<25 (+)	50 (-)	>	<25 (-)
*Nitzschia* sp.	>	>	>	>	25 (-)	50 (-)
*Surirella* sp.	NP	NP	NP	<25 (-)	<25 (-)	<25 (-)
*Tabellaria* sp.	>	>	>	<25 (-)	>	50 (-)
*Asterrionella* sp.	NP	NP	NP	NP	<25 (-)	<25 (-)
**Euglenophyceae**						
*Euglena* sp.	NP	NP	<25 (+)	>	>	25 (+)

> = no significant effect (NOEC ≥ 100 μg/L); NP = not present (taxa not present in any of the microcosms); significant increase (+) or decrease (-) compared to control.

### 3.2 Invertebrates

**Fig. 3:**
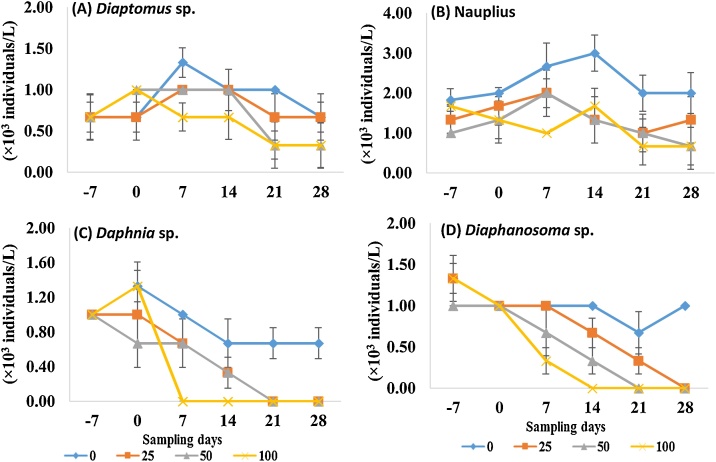
The population dynamics of the zooplankton taxa; (A) *Diaptomus* sp., (B) Nauplius, (C) *Daphnia* sp. and (D) *Diaphanosoma* sp. under the four concentrations (0, 25, 50 and 100 μg/L) of fenitrothion. Only the taxa that showed a significant response under treatment period for all sampling days are included.

A total of 8 zooplankton taxa were identified during the experimental period. The taxonomic group was dominated by Copepoda (3 taxa), followed by Cladocera (3 taxa) and Rotifera (2 taxa). Significant (p < 0.05) decrease was recorded in abundance for most of the zooplankton species from day 7 of the first fenitrothion exposure (Table 2). Four zooplankton taxa i.e. *Diaptomus* sp., Nauplius, *Daphnia* sp. and *Diaphanosoma* sp. were negatively affected from day 7 till the end of the experiment. Because 7−28-d NOEC (50 μg/L) was calculated for *Diaptomus* sp., 7-d NOEC (50 μg/L), 14−21-d NOEC (< 25 μg/L) and 28-d NOEC (25 μg/L) were calculated for Nauplius, 7−14-d NOEC (50 μg/L) and 21−28-d NOEC (< 25 μg/L) were calculated for *Daphnia* sp., and 7−28-d NOEC (50 μg/L) was calculated for *Diaphanosoma* sp.; while *Cyclops* sp. was negatively affected from day 21 onwards (NOEC of 25 μg/L) (Table 2; Fig. 3). The lower abundance of zooplankton in this study might be due to toxic nature of fenitrothion to zooplankton.

**Table 2:** No Observed Effect Concentrations (NOECs) for zooplankton and macroinvertebrates taxa expressed in terms of nominal single-dose of sumithion concentrations (μg/L) measured on each sampling day (One-way ANOVA; p < 0.05).

Endpoints	Sampling days					
	−7	0	7	14	21	28
**Copepoda**						
*Cyclops* sp.	>	>	>	>	25 (-)	<25 (-)
*Diaptomus* sp.	>	>	50 (-)	50 (-)	50 (-)	50 (-)
Nauplius	>	>	50 (-)	<25 (-)	<25 (-)	25 (-)
**Rotifera**						
*Brachionus* sp.	>	>	>	<25 (-)	<25 (-)	<25 (-)
*Keratella* sp.	>	>	>	<25 (-)	<25 (-)	<25 (-)
**Cladocera**						
*Moina* sp.	>	>	>	25 (-)	<25 (-)	<25 (-)
*Daphnia* sp.	>	>	50 (-)	50 (-)	<25 (-)	<25 (-)
*Diaphanosoma* sp.	>	>	50 (-)	50 (-)	50 (-)	50 (-)
**Molluscs**						
*Melanoides tuberculatus* (adult)	>	>	>	>	>	>
*Melanoides tuberculatus* (juvenile)	>	>	<25 (-)	<25 (-)	<25 (-)	<25(-)
*Viviparus bengalensis*	>	25(-)	25(-)	<25 (-)	<25 (-)	25 (-)
*Lamellidens marginalis*	>	>	50 (-)	>	25 (-)	25 (-)
**Annelid**						
*Tubifex tubifex*	>	>	50 (-)	>	<25 (-)	50 (-)
**Insects**						
*Notonecta* sp.	>	>	<25 (-)	<25 (-)	<25 (-)	<25 (-)
*Gerris* sp.	>	>	<25 (-)	<25 (-)	<25 (-)	<25 (-)
*Ranatra linearis*	>	>	<25 (-)	<25 (-)	<25 (-)	<25 (-)
Chironomid larvae	>	>	<25 (-)	<25 (-)	<25 (-)	<25 (-)

> = no significant effect (NOEC ≥ 100 μg/L); significant decrease (-) compared to control.

Unfortunately, the toxicity data (e.g. NOEC value) for the affected zooplankton taxa could not be found in open literature, and therefore the direct comparison is impossible. However, a study by Damasio et al. [43] calculated the 48-h IC50 value of fenitrothion for *Daphnia magna* as 1.87 μg/L, which is about 26 times lower than the NOEC value we calculated for *Daphnia* sp. (7-d NOEC = 50 μg/L). Leboulanger et al. (2011b) estimated the 48-h EC_50_ of fenitrothion for one of the copepods *Mesocyclops* sp. (1840 μg/L), which is approximately 18 times higher than we calculated for *Cyclops* sp. (7-d NOEC = >100 μg/L). In our study, the Rotifer *Brachionus* sp. and *Keratella* sp. were negatively affected from day 14 of the first exposure onwards with a consistent NOEC value of < 25 μg/L. Lv et al. [14] found less toxicity of fenitrothion on *Brachionus calyciflorus* than our study because they calculated a 4-d NOEC value of 1000 μg/L, which is several times higher than we reported in our study. Marcial et al. [13] reported similar range of results for another species of rotifer *Brachionus plicatilis* when fenitrothion was used as test compound. However, the variations in the toxicity of fenitrothion may be due to differences of species tested and formulation variation of chemicals [44].

**Fig. 4:**
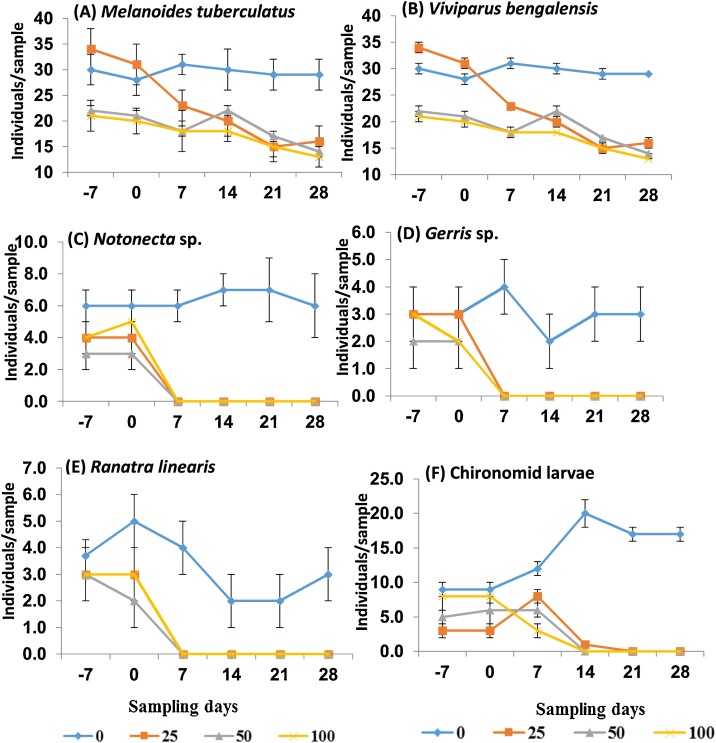
The population dynamics of the macro-invertebrate taxa: (A) *Melanoides tuberculatus* (juvenile), (B) *Viviparus bengalensis*, (C) *Notonecta* sp., (D) *Gerris* sp., (E) *Ranatra linearis* and (F) Chironomid larvae under the four concentrations (0, 25, 50 and 100 μg/L) of fenitrothion. Only the taxa that showed a significant response under treatment period for all sampling days are included.

There were 9 macro-invertebrates identified in the present study. The most abundant taxonomic group was Insecta (5 taxa), followed by Mollusca (3 taxa) and Annelida (1 taxa) (Table 2). All taxa belonging to Mollusca were negatively affected from day 7 at different concentrations of fenitrothion except *Melanoides tuberculatus* (adult). Results did not show consistent significant decrease for *Tubifex tubifex* for all sampling days during treatment period (Table 2). Univariate analysis showed significant decrease in abundance values (p < 0.05) for all identified insects (i.e. *Notonecta* sp., *Gerris* sp., *Ranatra linearis* and Chironomid larvae), because they were negatively affected at all treatments as compared to control for all sampling days during the treatment period (NOEC = < 25 μg/L) (Table 2; Fig. 4). It has been reported that combined exposure of neonicotinoid, organophosphate and herbicide caused oxidative injury in zebrafish [45]. Forcella et al. [18] observed a significant effect of fenitrothion on AChE inhibition of *Chironomus riparius* when exposed to different fenitrothion concentrations (0−100 μg/L). Almost similar degree of the AChE inhibition of *C. riparious* exposed to fenitrothion was observed by Choi et al. [46], but they also did not calculate any threshold values (e.g. NOEC, EC_50_, etc.).

### 3.3 Water quality parameters

Except on day 28 (NOEC = 25 μg/L), no significant effect was recorded on dissolved oxygen. No significant effects were observed for free CO_2_ and nitrate for any of the sampling days during the whole experimental period. A significant decrease was observed for pH on day 14. Total alkalinity levels decreased significantly for three consecutive sampling days (from day 7 to day 21), while phosphate concentrations significantly decreased on day 14 onwards in the treatment period (Table 3). The limited variation in water quality parameters in this study might be due to continuous use of aerator in the system. Unfortunately, we have not found any relevant literature contrasting our findings.

**Table 3:** No Observed Effect Concentrations (NOECs) for water quality parameters and Organic Matter Decomposition (OMD) expressed in terms of nominal single-dose of sumithion concentrations (μg/L) measured on each sampling day (One-way ANOVA; p < 0.05).

Water quality Parameters	Sampling days					
	−7	0	7	14	21	28
Dissolved oxygen	>	>	>	>	>	25 (-)
pH	>	50 (-)	>	50 (-)	>	>
Total alkalinity	>	>	50 (-)	25 (-)	<25 (-)	>
Free CO_2_	>	>	>	>	>	>
Nitrate	>	>	>	>	>	>
Phosphate	>	>	>	50 (-)	25 (-)	25 (-)
OMD	ND	ND	>	<25 (+)	>	>

> = no significant effect (NOEC ≥ 100 μg/L); significant increase (+) or decrease (-) compared to control; ND = not determined.

### 3.4 Organic matter decomposition

**Fig. 5:**
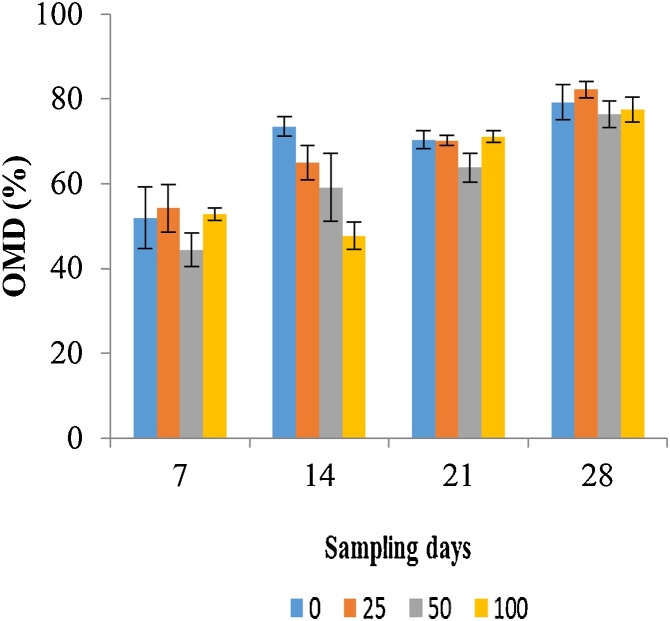
Organic matter decomposition (OMD) of banana leaves in different sampling days under four concentrations (0, 25, 50 and 100 μg/L) of fenitrothion.

In the present study, decomposition rates of banana leaves were 52 %, 74 %, 70 % and 79 % on day 7, 14, 21 and 28, respectively in the control group. Decomposition of banana leaves increased slightly over time during the treatment period. The statistical analysis, however, did not show a consistent significant increase except on day 14 of the first fenitrothion exposure (NOEC = <25 μg/L) (Table 3; Fig. 5). One of the earlier studies showed that there was no significant effect of one the neonicotinoid pesticide imidacloprid on the decomposition of banana leaves in their microcosm study [33].

## 4 Conclusion

This is the first report in assessing the toxicity of fenitrothion on structural and functional endpoints of freshwater microcosms. The present study revealed significant effects of fenitrothion on the abundances of most of the zooplankton, macro-invertebrate and some phytoplankton taxa except organic matter decomposition. We derived safe environmental concentrations of fenitrothion for different taxa through the derivation of NOECs, which would be useful for future ecological risk assessment of sub-tropical aquatic ecosystems. In the present study, as we observed several taxa were sensitive to even the lowest concentration of fenitrothion, more acute and chronic investigations are recommended including more species when exposed to < 25 μg/L of fenitrothion.

## Data availability statement

The corresponding author [Md. Shahjahan] is responsible to provide the supporting data for the findings of this investigation.

## Authors’ contributions

Mohammad Shadiqur Rahman conducted research and collected data; Kizar Ahmed Sumon drafted the manuscript; Md. Jasim Uddin edited manuscript; Md. Shahjahan designed and supervised the experiment, and edited the manuscript.

## Declaration of Competing Interest

The authors declare that they have no known competing financial interests or personal relationships that could have appeared to influence the work reported in this paper.
